# Strategies for Mitigating Phosphoric Acid Leaching in High-Temperature Proton Exchange Membrane Fuel Cells

**DOI:** 10.3390/molecules29184480

**Published:** 2024-09-20

**Authors:** Zhongming Xu, Nanjie Chen, Sheng Huang, Shuanjin Wang, Dongmei Han, Min Xiao, Yuezhong Meng

**Affiliations:** 1 The Key Laboratory of Low-Carbon Chemistry & Energy Conservation of Guangdong Province, State Key Laboratory of Optoelectronic Materials and Technologies, School of Materials Science and Engineering, Sun Yat-sen University, Guangzhou 510275, China; xuzhm26@mail2.sysu.edu.cn (Z.X.); chennj23@mail2.sysu.edu.cn (N.C.); huangsheng3699@163.com (S.H.); wangshj@mail.sysu.edu.cn (S.W.); mengyzh@mail.sysu.edu.cn (Y.M.); 2 School of Chemical Engineering and Technology, Sun Yat-sen University, Zhuhai 519000, China; 3 Institute of Chemistry, Henan Provincial Academy of Sciences, Zhengzhou 450000, China; 4 College of Chemistry, Zhengzhou University, Zhengzhou 450001, China

**Keywords:** fuel cell, high-temperature polymer electrolyte membrane, membrane electrode assembly, PA leaching, durability

## Abstract

High-temperature proton exchange membrane fuel cells (HT-PEMFCs) have become one of the important development directions of PEMFCs because of their outstanding features, including fast reaction kinetics, high tolerance against impurities in fuel, and easy heat and water management. The proton exchange membrane (PEM), as the core component of HT-PEMFCs, plays the most critical role in the performance of fuel cells. Phosphoric acid (PA)-doped membranes have showed satisfied proton conductivity at high-temperature and anhydrous conditions, and significant advancements have been achieved in the design and development of HT-PEMFCs based on PA-doped membranes. However, the persistent issue of HT-PEMFCs caused by PA leaching remains a challenge that cannot be ignored. This paper provides a concise overview of the proton conduction mechanism in HT-PEMs and the underlying causes of PA leaching in HT-PEMFCs and highlights the strategies aimed at mitigating PA leaching, such as designing crosslinked structures, incorporation of hygroscopic nanoparticles, improving the alkalinity of polymers, covalently linking acidic groups, preparation of multilayer membranes, constructing microporous structures, and formation of micro-phase separation. This review will offer a guidance for further research and development of HT-PEMFCs with high performance and longevity.

## 1. Introduction

**Figure 1:**
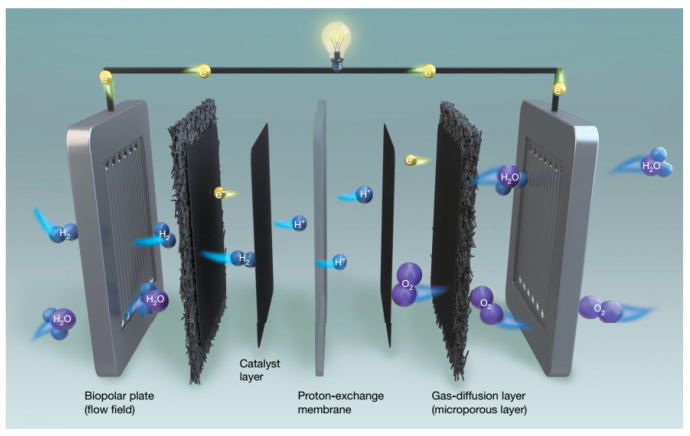
The operating principle of PEMFCs. Reproduced with permission from ref. [4]. Copyright 2021, Springer Nature.

The generation of clean electricity has become a prerequisite for sustainable development in modern society, increasing the attention paid to the energy transition path focused on hydrogen because of its potential to reduce greenhouse gas emissions and promote sustainable development [1]. PEMFCs, as a clean and efficient technology that directly converts the chemical energy of fuel into electricity, have garnered significant interest due to their remarkable performance across a wide range of temperatures, high efficiency, rapid startup, and extended stack life [2]. Traditional PEMFCs are mainly composed of a bipolar plate with channels, a PEM, a catalyst layer (CL), and a porous gas diffusion layer (GDL) [3]. The membrane electrode assembly (MEA) is composed of PEM, CL, and GDL (as shown in Figure 1), which is the site of the electrochemical reaction. Hydrogen is oxidized at the CL/PEM interface to produce electrons and protons at the anode. Protons are transported through the PEM to the cathode and react with oxygen in the air to form water.

PEMFCs can be categorized into low-temperature proton exchange membrane fuel cells (LT-PEMFCs) and HT-PEMFCs based on their operating temperature. The former typically operate below 80 °C, while the latter operates in the range of 100–200 °C. Both systems have their own strengths as well as challenges. The Nafion^®^ membranes used in LT-PEMFCs was jointly developed by DuPont in the 1960s [5] and has been successfully commercialized due to its superior physical and chemical stability advantages [6]. One of the limitations of such membranes, however, is that the proton conductivity of the membranes decreases dramatically at elevated operating temperatures (>100 °C) or in reduced-humidity environments (easy loss of water as a proton carrier). In spite of this, problems such as complex water/heat management system and CO poisoning in LT-PEMFCs are further limiting their development [7]. One possible strategy to mitigate the challenges encountered with PEMFCs involves the development of HT-PEMs capable of maintaining a certain level of proton conductivity in high-temperature environments [8]. The performance of HT-PEMFCs is improved at higher temperatures for the following reasons [9]: (1) the Pt catalyst employed in the cell has a tendency to adsorb impurities such as CO in the H_2_ below 80 °C, which reduces the overall performance of the fuel cell. However, the Pt’s affinity for carbon monoxide decreases when the temperature is above 160 °C, and its CO tolerance increases; (2) the overall performance of the fuel cell can be improved by accelerating the electrode reaction by raising the temperature; (3) this eliminates the need to consider the risk of water flooding the cell, as water exists in a gaseous state when the temperature is above 100 °C, greatly simplifying the water and heat management; (4) utilizing waste heat for cogeneration is easier at higher temperatures.

HT-PEMs, as the core component of HT-PEMFCs, play the most critical role in the performance of fuel cells. The majority of current reports on HT-PEMs research focus on the preparation of products with high performance, good thermal, mechanical, and chemical stability, and long durability, while reducing the overall cost of HT-PEMFCs [10,11]. The HT-PEMs can be divided into two parts: the membrane substrate material and the proton carrier. Polybenzimidazole (PBI) is the most commonly reported substrate material for HT-PEMs and can provide certain proton conductivity at high temperatures and has excellent chemical resistance, high mechanical strength, and thermal-oxidative stability above 80 °C [12,13]. In addition to PBI, polymers with basic groups such as polyether ketone, polysulfone, and polyphenylene oxide have also been reported for HT-PEMs applications [14,15,16,17]. The properties of polymer substrates have a significant impact on the membrane’s mechanical strength, heat resistance, and aging resistance. The vast majority of reported HT-PEMs use PA as the proton carrier for proton transfer [18]. In this system, in addition to the advantages of medium acidity, good thermal stability, and low vapor pressure [19,20], PA is particularly capable of self-ionization in the absence of water at high temperatures, with an ionization degree only slightly lower than that of H_2_SO_4_ [21,22]. It is capable of reacting with basic sites on basic polymers to form an acid–base composite polymer system, which in turn increases the proton conductivity of this type of PEMs [23,24] and solves the problem of the relatively low intrinsic proton conductivity of most basic polymer membranes [9]. Consequently, PA can be employed as a non-volatile proton carrier to entirely substitute for water in proton conduction in high-temperatures, low-humidity, or even anhydrous conditions [22].

Although PA-doped membranes have showed satisfactory proton conductivity in high-temperature and anhydrous conditions and significant advancements have been achieved in the design and development of HT-PEMFCs based on PA-doped membranes, the persistent issue of HT-PEMFCs caused by PA leaching remains a challenge that cannot be ignored. The following sections will provide a concise overview of the proton conduction mechanism in HT-PEMs and the leaching mechanism of PA and focus on reviewing the latest progress in reducing PA leaching, as well as methods to extend the life of HT-PEMFCs, and proposing new prospects for future research directions.

## 2. Mechanism of Proton Conduction and Leaching of PA of HT-PEMs

### 2.1. Proton Conduction Mechanisms

**Figure 2:**
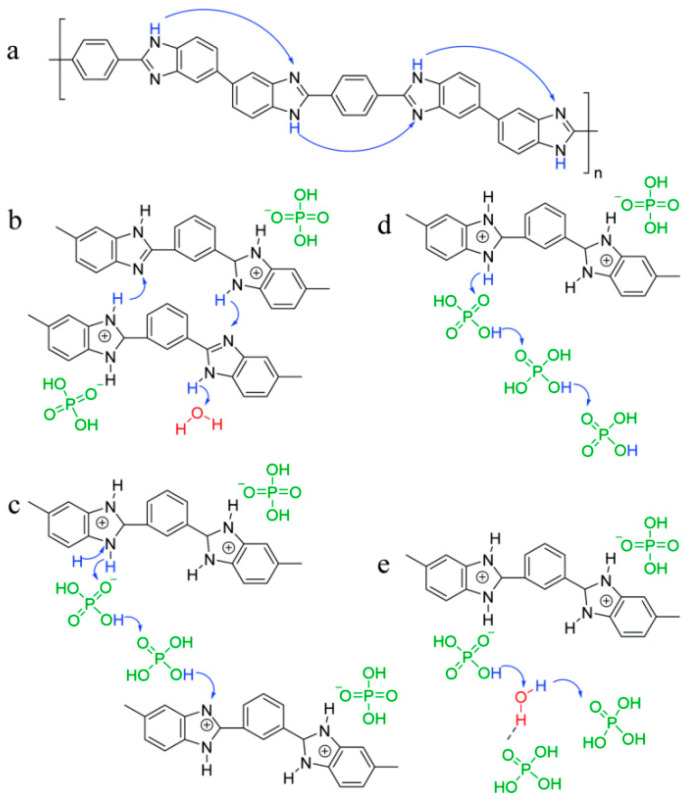
Chemical structure of (**a**) PBI, (**b**) H_3_PO_4_ protonated PBI, (**c**) proton transfer along acid–BI–acid, (**d**) proton transfer along acid–acid, (**e**) proton transfer along acid–H_2_O. Reproduced with permission from ref. [30]. Copyright 2021, Royal Society of Chemistry.

Proton conduction mechanisms are typically classified into two categories: the Vehicular mechanism and the Grotthuss mechanism [25]. The Vehicular mechanism is based on the proton binding to the proton “vehicle” (usually H_3_O^+^) and moving in a directional way from the anode to the cathode through the microstructure of the membrane to achieve proton conduction [26]. The second and faster mechanism is the Grotthuss mechanism, which relies on the hydrogen bonding network in the membrane, allowing the proton to be transferred from one “vehicle” to another by means of the stretching vibration of the hydrogen bond, while the original “vehicle” regains the ability to accept a new proton after the proton is transferred [27]. Von Grotthuss noted that when a proton interacts with a water molecule, it temporarily transforms into H_3_O^+^. At that instant, another proton separates from the original H_3_O^+^. A similar mechanism is shown to be involved in the Grotthuss chain between PA and the ion pair H_4_PO_4_^+^/H_2_PO_4_^−^ when PA is present [28]. Therefore, it is generally accepted that proton transfer in PA-doped PBI membranes mainly occurs through the Grotthuss mechanism, as shown in Figure 2 [29,30].

In PA-doped PBI membranes, the originally absorbed PA molecules are bound to the benzimidazole ring by Lewis basic N atoms (–N=) in the imidazole (Im) ring through acid–base interactions to form bound acids [31]. Further doping of PA gives free PA, but these free acids have a weak interaction with the polymer and are more easily washed out by water. It has been shown that when the amount of doped PA is low, protons mainly undergo the Grotthuss mechanism between N–H^+^ and H_2_PO_4_^−^; with further doping of PA, the number of protons transferring between H_4_PO_4_^+^ and H_3_PO_4_ molecules increases and its proton conductivity increases significantly [32]. Consequently, it is of vital significance to construct a network of N–H^+^···H_2_PO_4_^−^, H_3_PO_4_···H_2_PO_4_^−^, and/or N–H^+^···H–OH chains in order to achieve a high conductivity [33,34].

### 2.2. PA Leaching Mechanism

For proton carriers, the issue of how to control their doping and how to retain them in the membrane materials for a long time are essential considerations. The team of Hori et al. [35] tested the stability of commercial PBI membranes at 200 mA cm^−2^ and 190 °C and found that the output voltage of the cell dropped dramatically within 1000 h and that the internal resistance of the membrane increased dramatically, mainly due to the leaching of PA. It has been reported in the wider literature that the PA in the membrane can be divided into “bound PA” and “free PA” [36], and the second is bonded to the polymer by hydrogen bonding, which is the main carrier for proton transfer, but its weak force leads to the inevitable leaching of PA molecules in the operating HT-PEMFCs [34,37]. Not only does this lead to a sharp drop in cell output voltage, as Hori’s team found, but membranes with severe PA leaching can also lead to degradation and corrosion of fuel cells components, further reducing cells performance and decreasing cells durability [38,39,40]. Research into the mechanism of PA leaching has also been conducted for decades, and the most prominent mechanisms can be broadly classified into the following categories: (i) carrying over by water, (ii) evaporation of PA, and (iii) other factors.

#### 2.2.1. Carrying over by Water

Around 1980, Mori et al. [41] reported that PA molecules are transported to the cathode and anode during operation of HT-PEMFCs, leading to a decrease in PA in the membrane. The assembly pressure and capillary force within the membrane electrode assembly will lead to the migration and redistribution of PA molecules to the catalyst layer in the membrane, thus creating the conditions for the formation of the “solid–liquid–gas” electrochemical three-phase reaction interface [42], which is conducive to improving the performance of HT-PEMFCs. However, due to the water solubility and mobility of PA molecules, this also provides an opportunity for the water or water vapor generated from the reaction at the cathode to combine with the PA molecules and thus carry away the PA [43,44]. Simulations have been reported to show that free PA in the membrane is able to form stronger bonds with water molecules [45], especially at low to medium temperatures (<100 °C) and under the high humidity conditions that occur during cell startup and shutdown, leading to severe acid losses [46]. A type of PA leaching caused by a breakdown of the balance between PA and water was reported by Lee’s team [45]. They suggested that within a given volume of membrane material, when the PA and water within the basic polymer exceed a certain balance level, this leads to leaching of the PA from the polymer. Therefore, increasing the force between the PA and the membrane material is a viable strategy when considering how to reduce the leaching of PA, as more PA will be immobilized in the MEA and will be more difficult for water to carry away.

#### 2.2.2. Evaporation of PA

Although the acid vapor pressure of PA is very low, the acid loss due to PA evaporation when HT-PEMFCs are operated at high gas flow rates is still noteworthy. Yu et al. [47] reported in 2006 that they found the cathode-side PA leaching at 190 °C to be 10 times higher than that at 80 °C and 160 °C. Another study reported that the acid content in the gas phase was about 0.7 mbar and 3 mbar at 160 °C and 190 °C, respectively [48], when the HT-PEMFCs were worked at operating temperatures. Søndergaard et al. [49] reported that when the current density of the cell was set to 200 mA cm^−2^ and the stoichiometric amount of H_2_/Air was 1.2/2.0, the above saturated acid vapor pressure values at 160 °C and 190 °C could be converted to acid loss rates of 3.6 and 19 mg m^−2^ s^−1^, respectively [50]. It can be seen that the leaching of PA by evaporation cannot be ignored at temperatures above 160 °C.

#### 2.2.3. Other Factors

Lin et al. [51] found that external pressure may cause PA to extrude out of the MEA, leading to continued PA migration to locations such as the GDL and bipolar plate. While PA migrates to the bipolar plate, it may lead to corrosion of the bipolar plate material, thereby increasing the porosity of the plate [51]. This will in turn further increase PA leaching from the MEA to the outside. Eberhardt et al. [52] investigated the redistribution of PA under dynamic loading conditions in HT-PEMFCs by x-ray tomography (XTM). They found that under high-current-density operation, the PBI membrane exhibits PA migration from the cathode to the anode, which is due to the negatively charged hydrogen phosphate anion carrying part of the ionic current. After migrating to the anodic CL layer, PA continues to diffuse in the direction of the flow field, a process that is not fully reversible, leading to the loss of acid.

## 3. Strategies to Reduce PA Leaching

As previously described, free PA molecules, which are the key carriers of proton transport in HT-PEMFCs, are easily lost due to acid evaporation [47,48,49] or carrying over by cathode-generated water [43,44,45]. It not only reduces the proton conductivity and increases the membrane resistance for electrochemical reactions but also corrodes the components of the fuel cells and degrades the performance [38,39,40]. To address this problem, various strategies have been employed recently to mitigate PA leaching, which can be roughly summarized into the following categories: (i) designing crosslinked structures; (ii) incorporation of hygroscopic nanoparticles; (iii) improving the alkalinity of polymers; (iv) covalently linking acidic groups; (v) preparation of multilayer membranes; (vi) constructing microporous structures; and (vii) formation of micro-phase separation.

### 3.1. Designing Crosslinked Structures

A typical strategy to mitigate PA leaching is the preparation of HT-PEMs with a cross-linked structure, which can not only substantially enhance the dimensional stability, mechanical properties, and plasticization resistance of the membranes [53,54,55] but also increase the hindrance towards PA penetration and thereby achieve the stability of proton conductivity in the membranes [56,57]. The types of crosslinked structures can be divided into the following [53,58]: (i) ionically cross-linked membranes and (ii) covalently cross-linked membranes.

**Figure 3:**
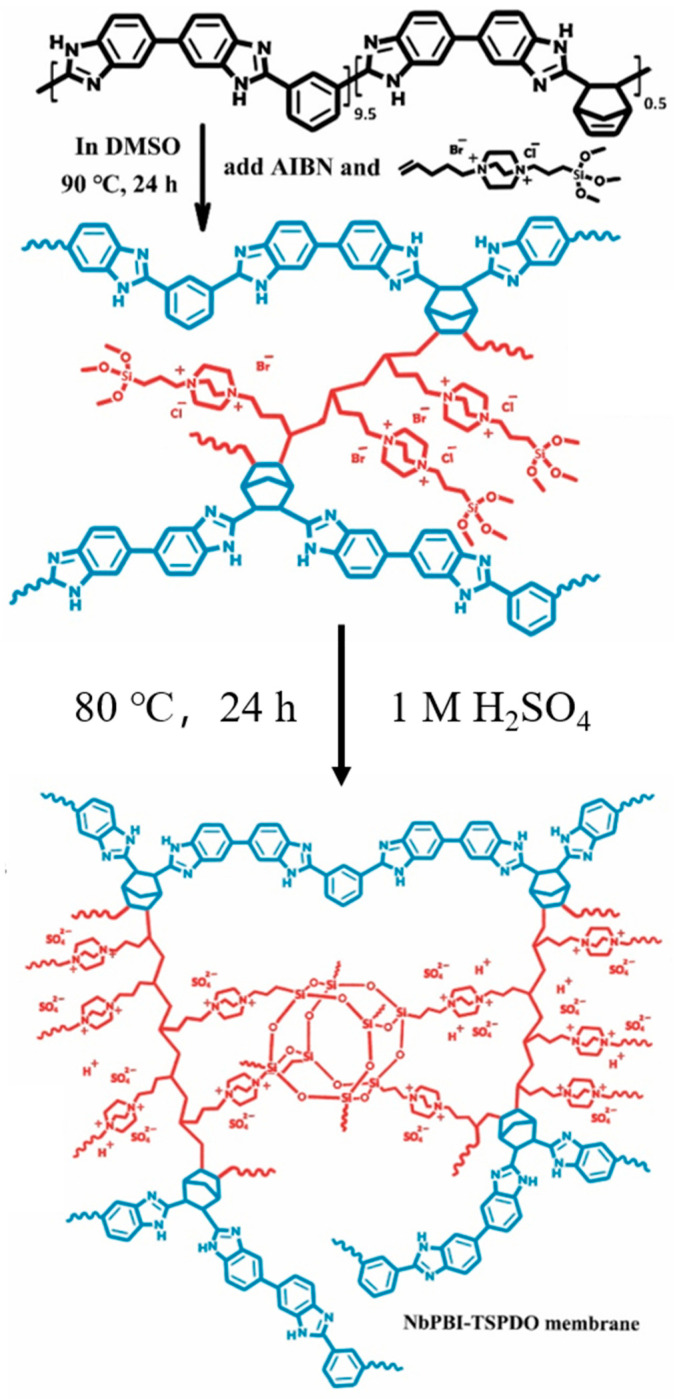
Preparation of NbPBI-TSPDO membranes. Reproduced with permission from ref. [59]. Copyright 2021, Elsevier.

Ionically cross-linked structures were designed through Lewis acid-base reactions via blending basic polymers with acidic polymers. In Liu’s work [59], they designed and prepared novel double cross-linked composite membranes from [TSPDO]BrCl and norbornene-type PBI (NbPBI) via in situ free radical polymerizations and in situ sol-gel reactions, as shown in Figure 3. The membranes are equipped with two cross-linked networks, which are the poly (ionic liquids) (PIL)-NbPBI (through Lewis acid–base reactions) and Si–O–Si networks (hydrolysis reaction of the trimethoxysilan groups). This cross-linked membrane demonstrated over 60% PA retention after 96 h at 80 °C and 40% RH due to the PIL-NbPBI cross-linked networks and the Si–O–Si networks.

Compared to ionically cross-linked structures, most studies have shown that the Im group in PBI is suitable for reacting with cross-link agents containing halogen [60,61,62] or epoxy groups [63,64] (N-substitution reaction) to form stronger covalently cross-linked membranes. Wang et al. [65] introduced a benzimidazole-grafted PBI membrane prepared from 2-chloromethyl benzimidazole (CMBelm) with Im groups. Then, a series of CPBIm-X cross-linked membranes were formed using 3-glycidoxypropyltrimethoxysilane (KH560) as a cross-linking agent. The Im groups of PBI could be N-substituted with the epoxy group of KH560, and its hydrolyses can form a cross-linked structure under acidic conditions, which could hinder the loss of PA and improve its stability. By measuring the retention of PA in each group (the membranes were placed at 70 °C and 60% RH), the PA leaching of CPBIm-X cross-linked membranes was lower than PBI due to the Si–O–Si cross-linked network structure in the matrix.

**Figure 4:**
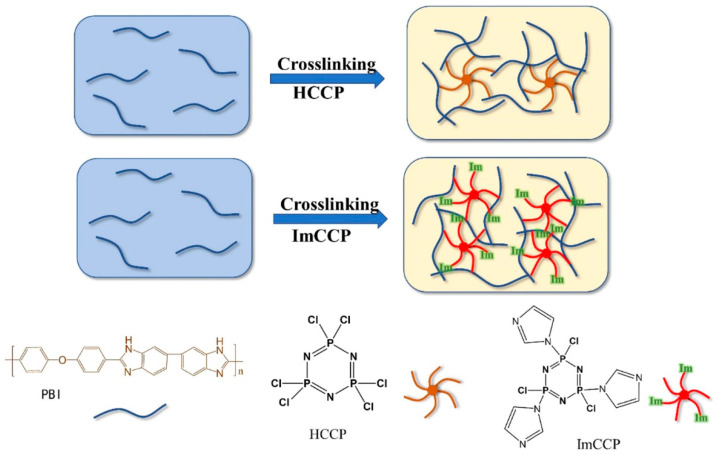
Schematic diagram of the preparation process of cross-linked PBI membranes. Reproduced with permission from ref. [54]. Copyright 2022, IOP Publishing.

Meng et al. [54] synthesized functionalized cross-linkers Im-chlorocyclotriphosphonitrile (ImCCP) from Im and hexachlorocyclotriphosphonitrile (HCCP) and successfully formed a series of cross-linked PBI membranes (PBI-HCCP membrane and PBI-ImCCP membrane) with excellent thermal stability and mechanical strength by using the N-substitution reaction of P-Cl on HCCP and ImCCP with the –NH site on PBI, respectively, as shown in Figure 4. Compared with PBI-HCCP membranes, PBI-ImCCP membranes shows higher gel content, lower acid doping level (ADL), and better dimensional stability and mechanical properties. The results revealed that densely cross-linked structure gives the cross-linked membranes better PA retention and mechanical properties than PBI membranes. The PA-doped PBI-ImCCP-20% membranes exhibited a tensile strength of 20 MPa, a PA retention of 80.5% after 96 h at 80 °C and 40% RH, and a proton conductivity of 54 mS cm^−1^ at 160 °C. The peak power density of the MEA with PBI-ImCCP-20% membranes reached 749 mW cm^−2^ at 160 °C without humidification.

**Figure 5:**
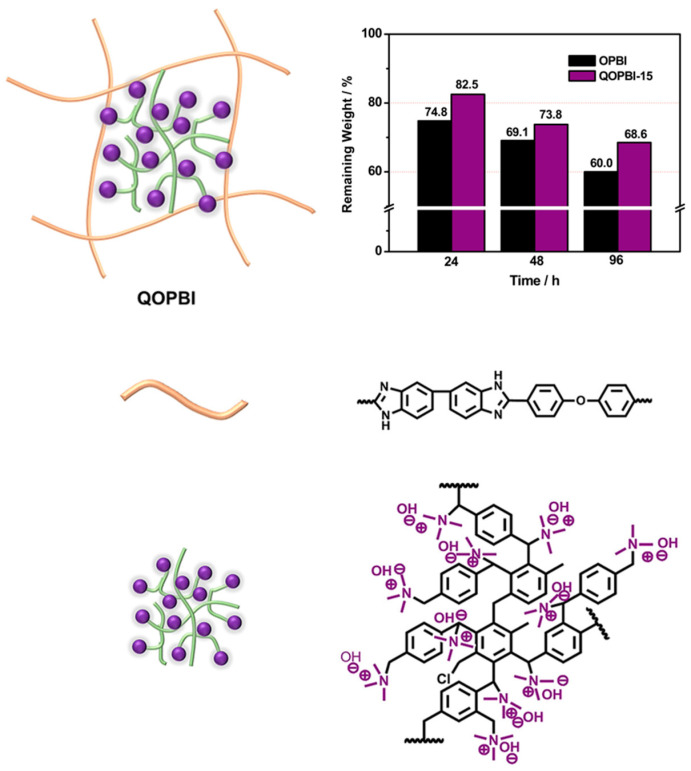
Chemical structure and the PA stability of QOPBI-X cross-linked membranes. Reproduced with permission from ref. [55]. Copyright 2020, Elsevier.

Xiao et al. [66] introduced polyaniline (PANI) with a 3D structure as a cross-linker to prepare a cross-linked PANI-poly(aryl ether benzimidazole) (OPBI) membranes. PA retention capacity testing (at 90 °C and 90% relative humidity) showed that the PA leaching of the PANI-OPBI membrane was 28.9% after 120 h, which is lower than that of OPBI. In addition to the experiments, the molecular dynamics simulation results indicate that the introduction of PANI can lead to the reduction in the PA leaching through the high intermolecular interaction energy of the aniline-biphosphate ion pair. A novel cross-linked membrane was recently reported by Wang’s team [55], achieved through the crosslinking of OPBI with bromomethylated poly(p-xylene) (Br-HPP), followed by immersion in a trimethy-lamine (TMA) solution to introduce a strongly basic quaternary ammonium (QA) group. This process involves a compound effect, beginning with the formation of a cross-linked structure via an amide-type bond, followed by the introduction of a QA group, thereby augmenting the membrane’s PA retention capacity through synergistic interaction with the QA group. Figure 5 shows that QOPBI-15 maintained more than 80% and 68.5% of its initial PA mass over 24 and 96 h, respectively, at 80 °C and 40% RH.

Sun et al. [67] fabricated cross-linked membranes using branched OPBI (BOPBI) with a rigid triazine structure and [BMIm][Br]. PA retention tests on cross-linked membranes with different PIL doping levels showed that these membranes still had about 50% PA retention after 240 h of testing at 80 °C and 40% RH. The BOPBI-PIL-40% cross-linked membrane was able to achieve a proton conductivity of 80.7 mS cm^−1^ even after the PA retention testing.

**Figure 6:**
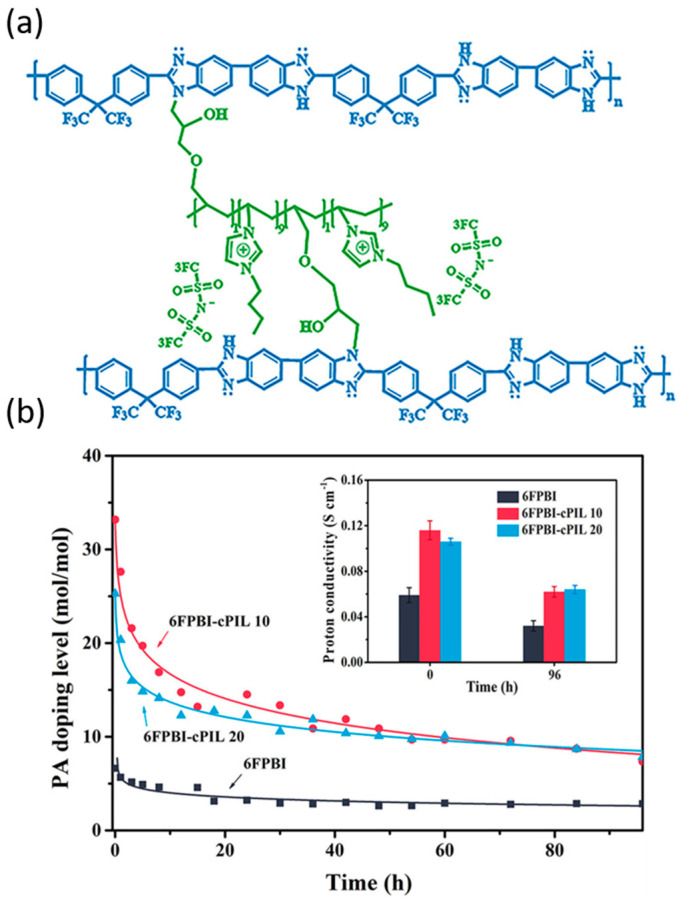
(**a**) Chemical structure of 6FPBI-cPIL; (**b**) PA uptake and proton conductivity stability of a series of 6FPBI-cPIL membranes over 0–96 h under conditions of 80 °C/40% RH. Reproduced with permission from ref. [68]. Copyright 2018, American Chemical Society (Open access).

Wang’s group [68] proposed a feasible strategy to prepare PIL containing both polymer cations and functional anions, through which they prepared a series of fluorinated PBI-cross-linked polymer ionic liquids (6FPBI-cPIL) composite cross-linked membranes. The incorporation of cPILs into the composite membrane leads a considerable quantity of ionic liquid moieties into the system, which possess ion–ion interactions with phosphate, resulting in a considerable amount of PA bound within the membrane, as shown in Figure 6. The 6FPBI-cPIL membranes exhibit enhanced PA retention capacity and long-term proton conductivity stability.

In spite of N-substitution, there are still other promising strategies for fabricating covalently cross-linked membranes for limiting PA leaching, like thermal curing [69], Friedel-Craft reactions [70], Diels-Alder [71] reactions, and reactions with other reactive groups [72].

Huang’s group [73] proposed the use of polyacrylamide (PAM) to prepare cross-linked HT-PEM. PAM is a highly hydrophilic and stable hydrogel that can ensure PA uptake and therefore has high proton conductivity. The PAM/PVA material is a typical product obtained by free radical polymerization, and the PAM/PVA semi-interpenetrating polymer network (IPN) membranes were obtained by using N, N′-(methylene) bisacrylamide (NMBA) as a cross-linking agent to crosslink PAM and PVA into a 3D IPN, which could not only improve the mechanical strength of PAM-based hydrogels but also increase the hindrance of PA penetration and reduce the leaching of PA.

In Jang’s [17] work, they established a cross-linked membrane (XTPPO), which was synthesized from poly (2,6-dimethyl-1,4-phenylene oxide)(PPO) and 1,7-octadiyne. The proton conductivity, mechanical stability, and PA retention ability can be significantly improved via the introduction of triazoles groups on the side chains of PPO. The PA leaching test was performed for a series of XTPPO, and the XTPPO-30-15 (where 35 refers to 35% degree of original brominating and 15 is the degree of cross-linkable azide groups) showed the highest PA retention at 80 °C and 50% RH for 1 h due to its higher degree of cross-linking, which increase the difficulty of water penetrating the PA-doped membranes.

It has been reported that thermal treatment of membranes at high temperatures leads to non-specific cross-linking of the polymer matrix [74], which has recently been shown to be an effective strategy for mitigating HT-PEM degradation [75]. Ossiander’s team [75] is committed to improving the durability of the cells through thermal post-curing of cross-linked PBI. In their recent work, cross-linked membranes were prepared from bisphenol A diglycidyl ether and meta-PBI, followed by thermal post-treatments. It was found that the resulting cross-linked membranes exhibited lower voltage decay rates. In Søndergaard’s work [76], they tested the performance of linear m-PBI and thermally cured m-PBI and found that the acid leaching rate of the cross-linked membranes was significantly reduced during the long-term operation of the cell.

### 3.2. Incorporation of Hygroscopic Nanoparticles

Incorporation of hygroscopic nanoparticles (e.g., silica (SiO_2_), zirconia (ZrO_2_), titanium dioxide (TiO_2_), graphene oxide (GO), and functionalized CNT into PA-doped HT-PEMs can improve both the proton conductivity and mechanical stability of the membranes and enhance their durability in MEA fabrication [77,78].

A novel PBI-BaZrO_3_ (PBZ) nanocomposite membrane was fabricated by Hooshyari’s team [77] via doping PBI with BaZrO_3_. The PBZ nanocomposite membrane exhibited significant proton conductivity and higher PA retention, presumably due to the hydrogen bond between the BaZrO_3_ and PA.

**Figure 7:**
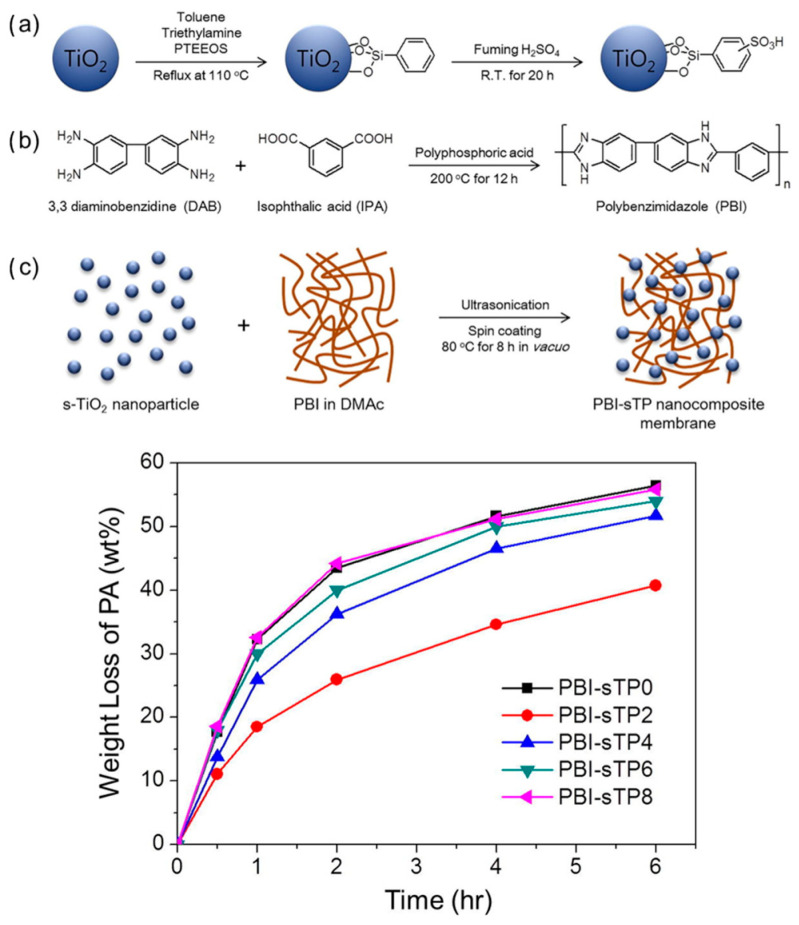
Schematic illustration of (**a**) the s–TiO_2_ synthesis, (**b**) synthesis of meta-PBI, (**c**) PBI-sTP nanocomposite membrane, and PA retention of PBI-sTP composite membrane. Reproduced with permission from ref. [82]. Copyright 2020, Elsevier.

Rajabi et al. [79] incorporated santa barbara amorphous-15 (SBA) mesoporous materials functionalized with polyamidoamine groups into the PBI/IL membranes and verified that the incorporation of SBA is beneficial for the stability of the cells via comparing the mass of PA retained by the membranes before and after doping in a water vapor environment, as well as by testing the proton conductivity of the treated membranes. Schonvogel et al. [80] proposed the incorporation of inorganic fillers, silicon carbide (SiC), into standard PBI-based Celtec^®^-P membranes. The composite membranes were found to exhibit lower acid loss during long-term operation via titration, the acid content excluded. This was attributed to the fact that more PA was retained within the membranes as the “bound PA”. Lobato et al. [81] prepared a series of PBI composite membranes with different TiO_2_ doping levels for HT-PEMs. After PA leaching tests on all membranes, it was found that the composite membranes with TiO_2_ content of 2 wt.% and 4 wt.% exhibited higher PA retention compared to the PBI membrane, reducing PA leaching by 4 times and 3 times, respectively. This is attributed to the positive effect of the formed aggregates in the composite membranes on the anchoring of PA. Among them, the composite membrane with 2 wt.% TiO_2_ showed the highest PA uptake and proton conductivity (reaching 43 mS cm^−1^). Recently, Lee et al. [82] modified TiO_2_ with silane coupling agents followed by sulfonation, and the introduction of sulfonate groups contributed to the enhancement of the proton transport mechanism and prevented the detrimental effects of agglomeration. By testing PA leaching in composite PBI membranes with different s–TiO_2_ doping levels, it was observed that the addition of approximately 2 wt.% s–TiO_2_ improved the retention of PA and reduced its leaching, and the loss of PA was reduced by about 33%, as shown in Figure 7.

Another inorganic hygroscopic nanoparticle popular among researchers is SiO_2_ or chemically modified SiO_2_ [83,84]. It has been reported that the addition of SiO_2_ can significantly improve the durability of commercial PBI membranes during HT-PEMFCs operation [85,86]. In situ X-ray diffraction revealed that new diffraction peaks of the phosphosilicate phase appeared in the membranes after doping with SiO_2_, leading to the immobilization of PA molecules in the form of a solid phase and thus alleviating the PA leaching [86]. Li’s team [87] prepared nanocomposite membranes by adding porous SiO_2_ nanoparticles into the cross-linked PBI membranes with additional Im groups in order to enhance the ability of membranes to hold the PA and alleviate the leaching of PA. The PA retention test showed that the PA leaching of c-PBI-20-SiO_2_-10 membranes was 36 wt.%, which was much lower than the 56 wt.% of Ph-PBI membranes. The increase in acid retention is supposedly due to the hygroscopicity of SiO_2_. Also, the porous structure of SiO_2_ provides further reservoirs for PA molecules.

Lysova et al. [88] prepared composite membranes of SiO_2_ and ZrO_2_ with 3,3-bis(p-carboxyphenyl)phtalide (PBI-O-PhT). The incorporation of SiO_2_ and ZrO_2_ allows for the adsorption of PA molecules on their surfaces, thereby retaining more acid in the PEM and reducing its leaching from the membrane. PA retention tests revealed that the proton conductivity of the non-doped PBI membrane decreased by two orders of magnitude after treatment with humid air flow, while the membranes obtained with oxide doping showed a decrease of less than one order of magnitude in proton conductivity after treatment. Additionally, compared to the ZrO_2_-doped membrane, the presence of SiO_2_ improved the retention capacity of PBI for PA.

**Figure 8:**
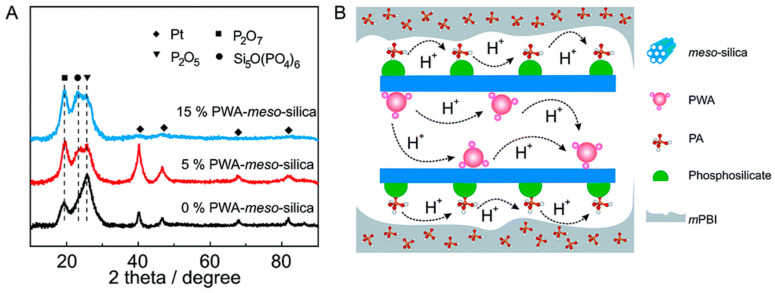
(**A**) XRD profiles of various PEMs with PWA-meso-SiO_2_ after durability test; (**B**) scheme of proton conduction paths through the attached and stabilized PA and PWA anchored inside the mesoporous channels of meso-silica at high temperatures. Reproduced with permission from ref. [86]. Copyright 2016, Royal Society of Chemistry.

In Aili’s work [86], they doped phosphotungstic acid (H_3_PW_12_O_40_·nH_2_O, PWA)-functionalized mesoporous SiO_2_ (PWA-meso-SiO_2_) into PBI membranes as a method to improve the durability of the fuel cells at high operating temperatures, and it operated stably for 2700 h at a load of 200 mA cm^−2^, with a low voltage degradation rate of 27 μV h^−1^. The XRD characterization of different PEMs after the durability test showed that there was a significant formation of P_2_O_5_ and P_2_O_7_ in the PA/PBI membranes, which indicated the instability of PA at 200 °C, as shown in Figure 8. However, a new peak at 23.5° was found in PWA-meso-SiO_2_-PA/PBI membrane, corresponding to the phosphosilicate phase Si_5_O (PO_4_)_6_, and the peak intensity increased with the increase in PWA-meso-SiO_2_ loading. Correspondingly, the peak intensity associated with P_2_O_5_ decreased significantly, demonstrating that the doping of PWA-meso-SiO_2_ improves the stability of PA in PEMs at high temperatures.

In Wang’s work [89], a SiO_2_-doped PA/PBI was designed by in situ formation of phosphosilicates. This in situ-formed SiO_2_/PA/PBI composite membranes showed a high conductivity of 53.5 mS cm^−1^ at 220 °C and excellent stability over 130 h, which was attributed to the form of the amorphous phosphorosilicate phase, inhibiting the evaporation and leaching of PA at high temperatures.

There is also a novel doping strategy of hygroscopic nanoparticles filled in the catalyst layer to limit further leaching of PA. Kim [90] added a small amount of Al_2_O_3_ (6 wt.%) into the catalyst layer. This is a new strategy to increase the durability and performance of HT-PEMFCs due to the formation of Al(H_2_PO_4_)_3_ from the reaction between Al_2_O_3_ and PA diffusing from the membrane.

**Figure 9:**
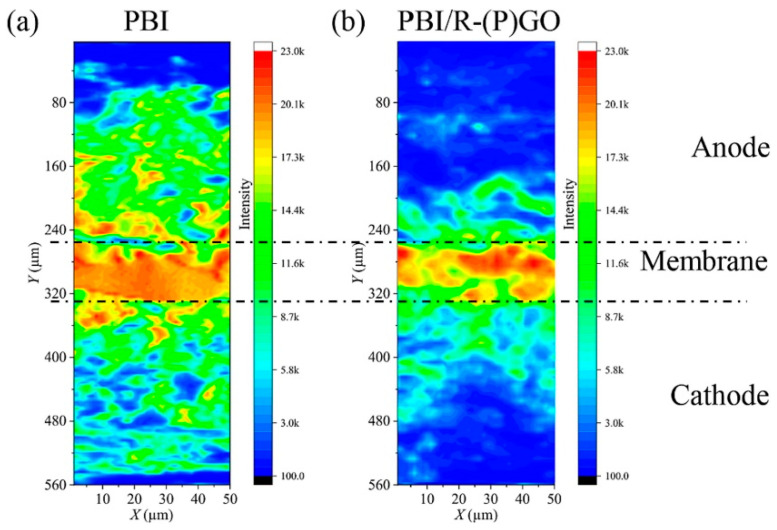
Synchrotron-based μ-XRF mapping of P distribution in MEA after 70 h AST. (**a**) MEA based on pure PBI membrane; (**b**) MEA based on PBI/R-(P)GO membrane. The color bar represents the scale of P intensities. Reproduced with permission from ref. [91]. Copyright 2023, Elsevier (Open access).

Doping HT-PEMs with 2D materials (graphene, functionalized graphene, and clay) with high surface area has likewise attracted interest. Sulfonated graphene oxide PBI composite membranes (PBI/s–GO) were proposed by Devrim’s team [78] via the casting technique. The incorporation of s–GO can promote the formation of proton transport channels in PBI, leading to a considerable level of proton conductivity. The PA uptake of the composite membranes increased with the levels of s–GO doping due to the fact that –SO_3_H in s–GO is a hydrophilic functional group. Compared with the cells assembled with PBI membranes, PBI/s–GO exhibited a lower voltage decay rate, with a performance loss of about 9% after a long-term stability test of 200 h, demonstrating that the presence of s–GO can form interactions with PA molecules and reduce the leaching of PA. Doping phosphonated graphene oxide (PGO) in membranes is also a promising strategy to improve the stability of HT-PEMFCs [91]. PGO was rapidly synthesized by one-step electrochemical exfoliation. PBI/(P)GO composite membranes showed excellent durability under an accelerated stress test (AST). As shown in Figure 9, the μ-XRF mapping of P distribution in the MEA of the PBI exhibits a severe leaching of PA, which migrates from the membrane to the electrodes on both sides and almost spreads over the entire MEA. In contrast, PA exhibits only a slight leaching in the MEA based on PBI/R-(P)GO membrane. A moderate amount of PA leaching is beneficial to reducing the dead zone between the membrane and catalyst and increasing the three-phase boundary area, which is conducive to the improvement of cell performance and stability.

Clay minerals with low cost and hygroscopicity are being explored for addition to membranes in HT-PEMFCs [92], offering the possibility of increasing proton channels and reducing hydrogen crossover. Muscovite (Mus, KA_l2_(Si_3_Al)O_10_(OH)_2_) has been reported to be an effective phosphate adsorbent with a smooth surface and high aspect ratio [93]. It can interact with polymer chains and PA molecules through the –OH groups, which is beneficial for proton conduction and PA retention. In Guo’s work [94], the PBI/Mus composite membrane with 2 wt.% Mus demonstrated the lowest voltage decay rate in the 70 h AST (0.226 mV h^−1^ at OCV, 1.042 mV h^−1^ at 600 mA cm^−2^, 1.298 mV h^−1^ at 1000 mA cm^−2^). Guo et al. [95] proposed a strategy for the preparation of composite membranes by using natural vermiculite (Verm) as the raw material to prepare exfoliated SiO_2_ nanosheets (E-SN) and sulphonated SiO_2_ nanosheets (S-SN), which were doped into the polymer electrolyte to fabricate P/SN, P/E-SN, and P/S-SN composite membranes, respectively. Characterization of these membranes showed promise in terms of ADLs, hydrogen permeability, and durability.

Lv et al. [96] prepared m-PBI-x wt.%/CeO_2_/g-C_3_N_4_ composite membranes by doping CeO_2_/g-C_3_N_4_ nanosheets in m-PBI. The incorporation of the nanosheets enhanced the absorption of PA and the formation the proton transport channels, resulting in a higher cell activity (540 mW cm^−2^) as well as higher durability, with a voltage decay rate of 0.188 mV h^−1^ (160 °C, 300 mA cm^−2^).

### 3.3. Improving the Alkalinity of Polymers

PA-doped HT-PEMs are actually “acid–base composite membranes”, which means that PA molecules are doped into the alkaline polymer electrolyte membranes and interact with alkaline sites. A desirable strategy is increasing the alkalinity of the polymer by introducing more or stronger alkaline groups into the polymer backbone to enhance the interaction between the PA molecules and the polymer [97], thus reducing the leaching of PA [66,98,99].

**Figure 10:**
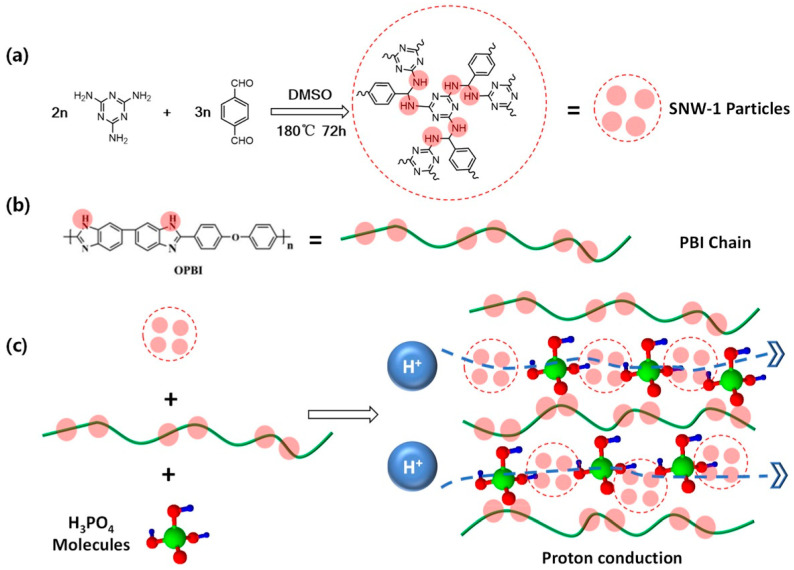
(**a**) Synthesis process of SNW-1. (**b**) Structure of PBI chain. (**c**) Schematic of proton conduction in PBI/x%-SNW-1 composite membranes. Reproduced with permission from ref. [100]. Copyright 2022, Elsevier.

Meng’s group synthesized a typical Schiff-base type porous polymeric network (SNW-1) with abundant N–H sites and blended it with PBI [100]. Consecutive proton transportation channels were constructed in the composite membrane due to the dense N–H sites and porous structure, which initially achieved a lower PA absorption (175.0%), a higher proton conductivity (114.0 mS cm^−1^ at 160 °C), a better cell performance (590 mW cm^−2^), and a higher proton conductivity stability (only 8.5% decrease in proton conductivity at 30 h) than those of PBI. This occurrence is attributable to the porous structure and numerous N–H sites of SNW-1, capable of anchoring PA molecules through both internal and external H-bond networks, as well as enclosing PA molecules in a porous structure (as shown in Figure 10). Furthermore, the relatively low PA uptake of PBI/30%-SNW-1 means fewer free acid molecules, which implies fewer “free acid” and more “bound acids”, which suggests the feasibility of reducing PA leaching and thus obtaining stable proton conductivity.

Luo et al. [101] prepared AmPBI-P3 membranes by grafting flexible side chains containing Im groups on aminopolybenzimidazole (AmPBI), which were tested for PA retention at 80 °C/40% RH, with PA retention approaching 80% at 48 h. After 240 h of PA retention testing at 160 °C without humidity, the proton conductivity only decreased by 14% after 240 h (still reaching 94.9 mS cm^−1^).

Lee [44] fabricated QA-biphosphate ion-pair-coordinated polyphenylene (PA-doped QAPOH) PEMs by Diels–Alder reaction [102]. They calculated the intermolecular interaction energies between PA and water, PA and PBI, and PA and QA, respectively, and the results showed that there was a strong interaction between PA and QA (151.7 kcal mol^−1^, much larger than that between PA and water). The stable ion pair can be fabricated via the alkaline QA groups and phosphate ions after PA-doping the PEMs. They also proposed that one of the main reasons for PA leaching in HT-PEMs is that the intermolecular interaction energy of PA with PBI is only slightly larger than that of PA with water (17.4 kcal mol^−1^ and 15.4 kcal mol^−1^, respectively). It is concluded that the formation of stable ion pairs effectively immobilizes PA within the membrane, providing more possibilities for reducing PA leaching.

Wang et al. [98] grafted long alkyl chains containing QA groups onto the main chain of PBI via the Menshutkin reaction [103]. The PBI-Sc-35 membranes achieved 80% retention of PA after 72 h exposed to 80 °C/40% RH, and the proton conductivity remained stable at 78 mS·cm^−1^ at 150 °C. This demonstrated that soft long side chains contribute to proton migration via heterocycles aggregation and hydrogen bond formation, and improving the polymer alkalinity is an effective way to enhance the PA retention capability.

In Zhang’s work [104], they synthesized HT-PEMs (PIT and PIB) with a lactam structure and without ether bonds and introduced glycidyl trimethyl ammonium chloride (GTA) side chains with QA and hydroxyl groups. The QA groups on the side chains are able to act as ion-pairing and acid–base interaction sites for PAs, and in addition, the presence of hydrophilic hydroxyl groups enhances the hydrogen-bonding interaction sites. Therefore, this structure makes a significant contribution to the PA retention and cell activity; the PA retention of PIT-GTA and PIB-GTA was as high as 79.2% and 83.6% after 26 h in a humid environment at 80 °C and 40% RH, and the cell activities were 464 mW cm^−2^ and 632 mW cm^−2^ in a single-cell performance test at 160 °C, respectively.

Bai et al. [105] designed a HT-PEM based on chloromethyl polysulfone (CMPSf) and Poly(vinyl pyrrolidone) (PVP). In this system, the QA group is generated by nucleophilic substitution of chlorine atoms with nitrogen atoms on PVP. The ion-pair interaction between the ammonium and PA results in a membrane with both high cell activity (729.4 mW cm^−2^) and good durability (110 h of non-decaying voltage at 150 °C and 520 mA cm^−2^). Wu et al. [106] prepared PILs with QA groups obtained with 1-vinylimidazole and tetrabromopentaery-thritol, which were introduced into OPBI to enhance the proton conductivity and PA retention capacity of HT-PEM. The single-cell performance of the OPBI -PIL- 20% (the PIL is 20 wt.% in HT-PEM) achieved a single-cell performance of up to 436.19 mW cm^−2^, and the voltage was maintained without degradation for 110 h in a long-term stability test at 160 °C, 100 mA cm^−2^.

Another approach was to incorporate small molecules containing acidophilic QA groups as crosslinkers into PBI membranes [107]. The tightly cross-linked structure provides the membrane with excellent mechanical strength, while the introduced QA groups form the ammonium-biphosphate ion-pair structure after PA doping. It provides an unexpected possibility for the membranes to retain the PA molecules, exhibiting a low voltage decay rate within 72 h at 160 °C with a back pressure of 200 kPa and a current density of 500 mA cm^−2^.

**Figure 11:**
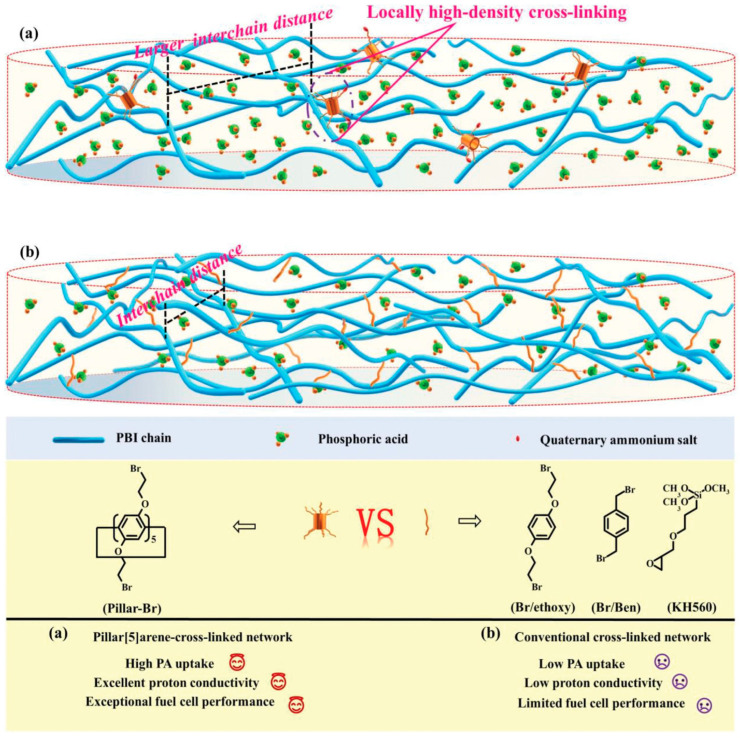
(**a**) Molecular structures and features of conventional and (**b**) pillar arene-based cross-linked networks. Reproduced with permission from ref. [109]. Copyright 2023, John Wiley and Sons.

Xiao’s team [108] reported a x-QPANI-OPBI membrane that was fabricated by introducing PANI with different QA group contents into OPBI. Leaching of PA was reduced due to the construction of both aniline-diphosphate ion-pairs and QA-diphosphate ion pairs. In Peng’s work [109], a locally high-density cross-linked PBI membrane based on pillar [110] arene bearing multiple alkyl bromide was constructed, as shown in Figure 11. The unreacted halides remaining on the column aryl hydrocarbon were converted to ammonium salts, which provided additional acidophilic sites for acid uptake and proton transport. OPBI-CL-Pillar-7% exhibits a voltage decay rate of only 0.0395 mV h^−1^ in a 200 h durability test.

**Figure 12:**
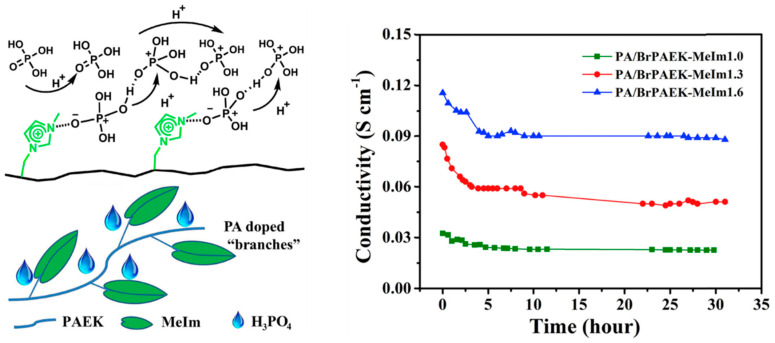
Schematic illustration of “frustrated” hydrogen-bond network and the proton conductivity of PA/BrPAEK-MeIm membranes at 160 °C for an extended time. Reproduced with permission from ref. [111]. Copyright 2018, Elsevier.

There is a potential strategy of grafting a nitrogen heterocyclic to aromatic polymers. The introduction of alkaline sites leads to an enhanced interaction between the polymer substrate and PA due to the stable conjugate nitrogen heterocyclic providing acid adsorption sites. Wang et al. [111] utilized four nitrogen heterocycles (pyridine, 1-methylimidazole, 1H-benzotriazole, and 3-amino-1,2,4-triazole) and modified poly (arylene ether ketone) (PAEK) as a polymer matrix for HT-PEMs (Figure 12). Due to the stronger alkalinity of Im, 1-methylimidazole-modified PAEK showed more effectively improved proton conductivity and stability of proton conductivity compared to the other three groups of modified PAEKs. The prepared BrPAEK-MeIm1.6 membrane (1.6 Im units per repeat unit) exhibited a proton conductivity of 91 mS cm^−1^ at 170 °C (with excellent stability over the next 25 h).

Xiao’s team [112] prepared a series of grafted PBI containing benzimidazole-type side pendants via N-substitution reactions. The introduction of Im groups led to higher ADLs, attributed to the additional basic benzimidazole groups increasing the intermolecular forces between the polymer framework and PA, further promoting PA uptake. After 120 h under extreme humid conditions, the g3-OPBI membrane showed very little acid loss, approximately 46% less than the original OPBI membrane.

In Jang’s work [113], six azole-containing membranes were prepared by side-chain functionalization of poly(phenylene oxide) with triazoles, benzimidazoles, and imidazoles, and they verified that two factors, N-substituent and pKa, have a great influence on PA retention and proton conductivity, which provides a potential key solution to solving the problem of PA leaching and developing high-performance and long-term-durable HT-PEMFCs.

### 3.4. Covalently Linking Acidic Groups

Contrary to introducing alkaline functional group to increase the bonded PA, another potential strategy is the direct incorporation of acidic groups with the possibility of conducting protons into the PEMs to achieve intrinsic proton conduction by immobilizing protonic groups [28]. The common organic acid groups with sufficient hydrolysis and oxidation resistance are –SO_3_H, –COOH, and –PO_3_H_2_. The weak acidity and low ionization degree of the –COOH group make it difficult to achieve the requirements of PEMFCs [114,115]. Consequently, more research has been focused on the –SO_3_H and –PO_3_H_2_ groups (Table 1).

Sana et al. [116] prepared PEMs based on a series of PBI composites using three acidic surfactant molecules (camphorsulfonic acid (CSA), p-toluenesulfonic acid (PTSA), and mono-n-dodecyl phosphate (MDP)). The experimental results showed that the leaching rates of PA for OPBI, OPBI/MDP-20%, OPBI/PTSA-20%, and OPBI/CSA-20% were 64%, 54%, 33%, and 27%, respectively, demonstrating the effectiveness of incorporation of the acidic surfactant in reducing the PA leaching. This can be attributed to the fact that the –PO_3_H_2_ or –SO_3_H group in the acidic surfactant facilitates the formation of strong hydrogen bonding interactions between the PA molecules and the polymer chains, thus enabling a greater number of PA molecules to be retained within the membrane material.

Liu et al. [117] incorporated hydrophilic and acidophilic SPOSS nanoparticles into high-molecular-weight aryl ether-based PBI with the assistance of sulfonated PAEK. The acid leaching was calculated under humid conditions. Compared with the original membranes, the acid loss of the PBI/SPAEK-SPOSS membrane was reduced throughout the experimental period and decreased with the increase in the SPOSS content, which was only 38% after 9 h of treatment for PBI/SPAEK-SPOSS-3%; in contrast, the acid loss was as high as 60% for Ph-PBI.

PA-doped sulfonated PBI membranes (s-PBI) were reported by Mader et al. [118]. They found that s-PBI membranes exhibited excellent proton conductivity (>100 mS cm^−1^ at elevated temperatures) and operating stability, with a voltage decay rate of only 30 μV h^−1^ at 160 °C and 200 mA cm^−2^ over 3000 h.

**Figure 13:**
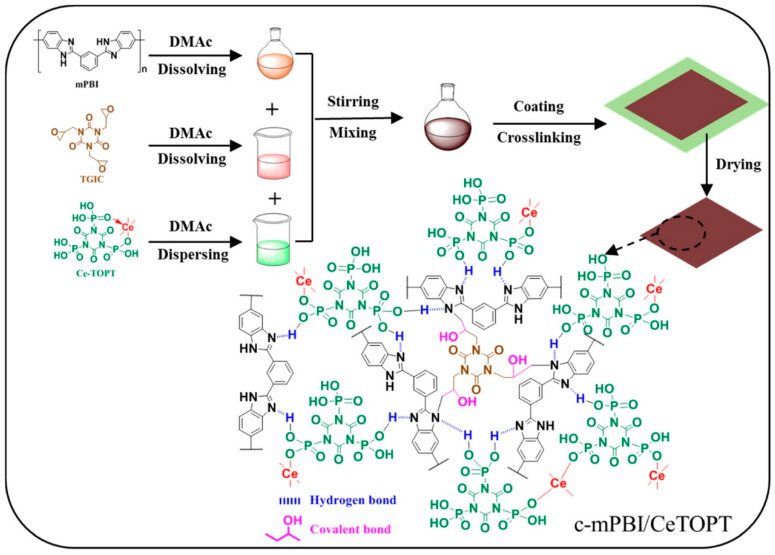
The synthesis and chemical structure of a c-mPBI/CeTOPT composite membrane. Reproduced with permission from ref. [124]. Copyright 2021, IOP Publishing (Open access).

In comparison to inorganic PA, the –PO_3_H_2_ groups are directly connected to the main chain or side chain of the polymer by a C–P bond. This eliminates the disadvantages of inorganic PA, such as high corrosiveness and ease of leaching [119,120,121]. Thus, PA can be replaced by organic compounds containing –PO_3_H_2_ groups [122,123]. Guo et al. [124] developed a strategy to overcome the acid leaching issue in PA-doped PBI membranes by synthesizing the cerium triphosphonicisocyanurate (Ce-TOPT) proton conductors and incorporating them into cross-linked PBI main chains (c-mPBI). The TOPT organic compound contains three –PO_3_H_2_ groups that can be complexed with highly valent metal ions (Ce^4+^/^3+^, Zr^4+^, Fe^3+^) in varied molar ratios, ensuring their insolubility in water (as shown in Figure 13). The organic–inorganic Ce-TOPT exhibited a high doping level in c-mPBI/CeTOPT membranes, enabling the membranes to have high proton conductivity at high temperatures and low relative humidity. At 180 °C, the conductivity of c-mPBI/CeTOPT (50) reached 125, 88.5, and 36.3 mS cm^−1^ under 100% RH, 50% RH, and anhydrous conditions, respectively. After 48 h of water washing, the proton conductivity loss of c-mPBI/CeTOPT (50) was only 4.6%.

The PBI-NH_2_-EPA membranes were fabricated by grafting ethyl phosphoric acid onto amino-modified PBI [125]. During PA retention testing in humid environments (at 70 °C and 60% RH for 240 h), PBI-NH_2_-EPA exhibited a higher level of ADL than the PBI membrane.

**Figure 14:**
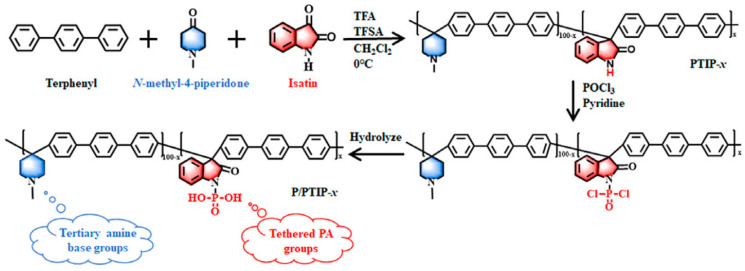
Synthesis of a bifunctional P/PTIP-x copolymer. Reproduced with permission from ref. [127]. Copyright 2024, John Wiley and Son (Open access).

Meng’s group [126] explored a strategy to achieve low acid leaching via replacing free PA with immobilized PA. They introduced phosphonated phenolformaldehyde (PPF) as an intrinsic proton conductor into PBI to construct proton transport channels in HT-PEM membranes. The PPF with immobilized PA not only achieves anhydrous proton conductivity at high temperature but also mitigates the detrimental effect of PA on membrane swelling. The power density of the PPF/50PBI membrane (with PA uptake less than 150%) reached 607 mW cm^−2^, and the voltage decay rate was only 12% in 50 h due to the excellent PA retention properties. This group also synthesized a bifunctional poly (p-terphenyl-co-isatin piperidinium) copolymer with tethered PA and intrinsic tertiary amine base groups (abbreviated as P/PTIP-x, shown in Figure 14) [127]. With PA doping, protons can not only transport through the hydrogen bond network formed by the free PA anchored by the tertiary amine base groups but also rely on the proton channel constructed by the ionic cluster formed by the tethered PA aggregation. Therefore, the bifunctional copolymers with lower PA uptake level (<100%) can display a prominent proton conductivity of 99 mS cm^−1^ and peak power densities of 812 mW cm^−2^ at 160 °C along with low leaching and high voltage stability, demonstrating that the design of intermolecular acid–base pairs can realize win-win demands between the mechanical robustness and electrochemical properties of HT-PEMs.

### 3.5. Preparation of Multilayer Membranes

Encapsulation membranes with multilayer structures have been extensively studied as they represent an important strategy for minimizing acid loss. By stacking different types of membranes, the PA molecules can be effectively trapped within the membrane, thus reducing acid loss (Table 2) [40,50,128,129].

Layered (Kevlar-CdTe-PA)_4_ membranes were synthesized by multiple repetitions of the spin-coating method using Kevlar nanofibers as the supporting backbone and CdTe nanocrystals as the medium connecting the PA and Kevlar nanofibers [130]. The layered distribution of components is expected to minimize fuel crossover and PA leaching to a certain level.

**Figure 15:**
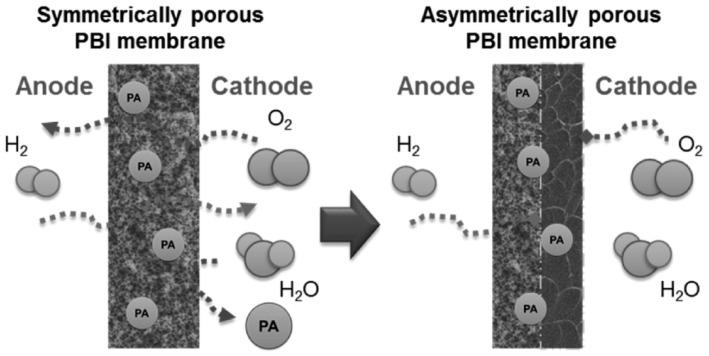
Schematic illustration of how an asymmetrically porous PBI membrane has the potential to avoid fuel crossover and acid leakage issues. Reproduced with permission from ref. [128]. Copyright 2016, Elsevier.

Porous membranes are capable of absorbing more PA but are also more susceptible to fuel crossover and PA leaching because the continuous channels allow gas and free PA to pass through on a microscopic level [60,131]. Cheng et al. [128] developed symmetric and asymmetric porous PBI membranes using the template leaching method, where the asymmetric structure consists of porous PBI on the anode side and dense PBI on the cathode, as shown in Figure 15. After 150 h of cell operation, it was found that the cell assembled with symmetric porous PBI membranes had a severely voltage decay rate (0.810 mV h^−1^). In contrast, dense PBI and asymmetric porous PBI membranes exhibited lower voltage decay during cell operation, 0.294 mV h^−1^ and 0.283 mV h^−1^, respectively, and these results suggest that porous PBI membranes with asymmetric porous have better durability for fuel cell operation.

Lu et al. [129] prepared a novel composite membrane by stacking two membranes with different porosity and functionality, i.e., microporous glass fiber (GMF) and polytetrafluoroethylene (PTFE), together. In this case, PTFE covers both sides of the GMF in order to minimize the loss of PA from the porous GMF and to provide good mechanical strength for the multilayer membrane structure. Finally, the proton conductivity loss of the GMF/mPTFE membrane after 1 h at 150 °C was only 25%, which is much lower than that of the single-layer GMF membrane (65%).

**Figure 16:**
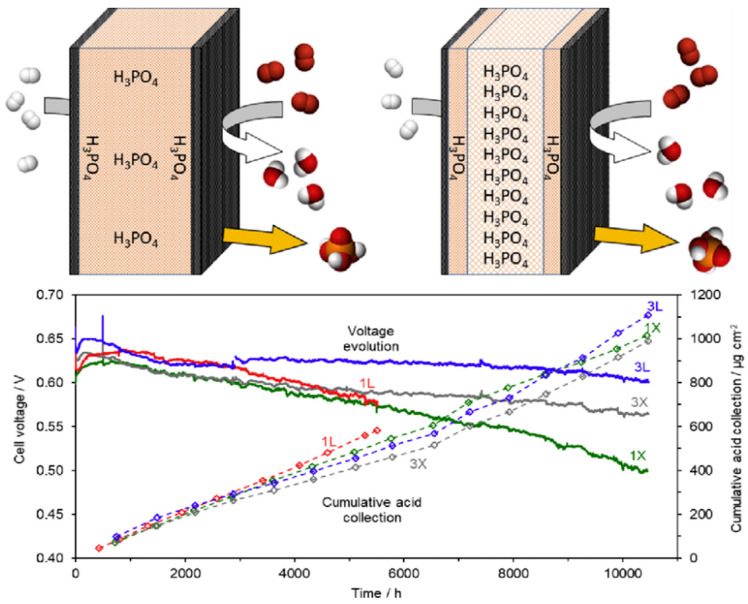
Schematic structure and fuel cell durability tests of the three-layer membrane structure prepared by Kannan. Reproduced with permission from ref. [40]. Copyright 2020, Elsevier.

Three-layer encapsulation structures based on PBI membranes were developed by Kannan [40]. The intermediate membrane layer is synthesized directly in polyphosphoric acid which serves as an acid reservoir [132], and the protective layers on both sides with relatively low PA adsorption are synthesized from PBI membranes with linear or crosslinked structures. The barrier layer is expected to limit the PA molecules from leaking out of the center layer, and the schematic structure is shown in Figure 16. Compared with the corresponding single-layer membrane fuel cells, the voltage decay rate of the three-layer membrane (much thinner than the former) fuel cells was significantly lower (average cell voltage decay rates are 14 μV h^−1^ and 2.3 μV h^−1^ for single-layer and three-layer membrane, respectively).

Wang et al. [133] also reported a novel leaf-like three-layer PEM for HT-PEMFC, with the porous PBI in the center acting as an acid reservoir to provide high proton conductivity while the dense PBI on both sides acted as a surface protection layer. The unique dense–porous–dense structure of the multilayer membrane leads to high PA uptake as well as good mechanical properties and high acid retention. HT-PEMFCs assembled with the three-layer PEM exhibited a voltage decay rate of only 0.730 mV h^−1^ in a 200 h test [134].

**Figure 17:**
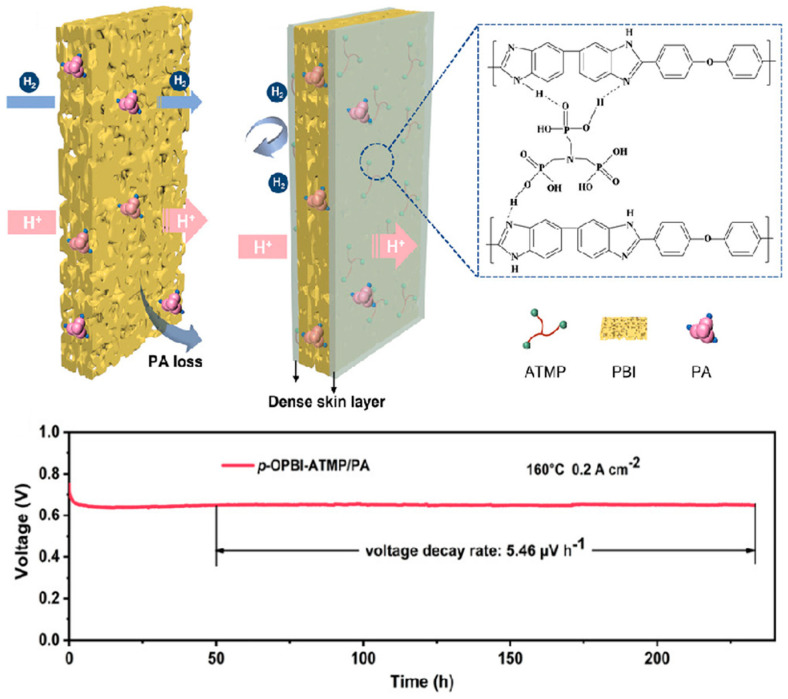
Schematic structure of p-OPBI-ATMP and performance tests for voltage decay rate. Reproduced with permission from ref. [135]. Copyright 2023, John Wiley and Sons.

Similarly, another porous PBI membrane with a dense double skin layer was recently reported by Lu [135]. By reacting the cross-linking agent amino tris (methylene phosphonic acid) with the porous PBI, thus allowing the formation of a dense PBI membrane of a certain thickness on the surface, this multilayer membrane (p-OPBI-ATMP) displays high proton conductivity along with high stability and low voltage attenuation rate, as shown in Figure 17.

### 3.6. Constructing Microporous Structures

The PA-doped porous PEMs exhibited enhanced proton conductivity due to the increased capacity to accommodate the PA molecules [136]. However, the large pore size of these membranes is not conducive to long-term retention of the PA, and the plasticization leads to a reduction in the membrane’s mechanical strength, which is not conducive to fuel cell operation [44,137]. A novel approach is to synthesize PEMs with interconnected nanoscale channels to adsorb PA molecules by capillary force. This enhances the interaction between the membrane and PA while reducing acid loss without compromising proton conductivity [138].

Guo et al. [139] prepared porous PES/PVP membranes with sub-micropores by template leaching using monodispersed SiO_2_ solid spheres (about 250 nm in diameter). In the PA-doped porous PES/PVP, the improved proton conductivity is the result of the cooperative proton transfers along both the “bonded acid” caged inside the pores and the “free acid” in the sub-micropores. Furthermore, the single cell shows almost no significant degradation during more than 150 h of testing, which is due to the well-preserved sub-micropores structure that helps in maintaining the concentration of both the “bonded acid” and the “free acid”.

Wang et al. [140] reported novel alloy membranes with a special intrinsic porous structure via doping the polymers of intrinsic microporosity (PIMs) with two different molecular weights into the OPBI matrix. The weight changes of PA-doped membranes were evaluated at 80 °C and 40% RH for different durations. Compared to the PA-doped OPBI membranes, the PA-doped alloy membranes with PIM-1 content less than 10 wt.% showed improved PA stability. The PA-doped L-10 membranes showed the best PA retention with a PA residual of 65.4 wt.% after 216 h of exposure to 40% relative humidity at 80 °C.

**Figure 18:**
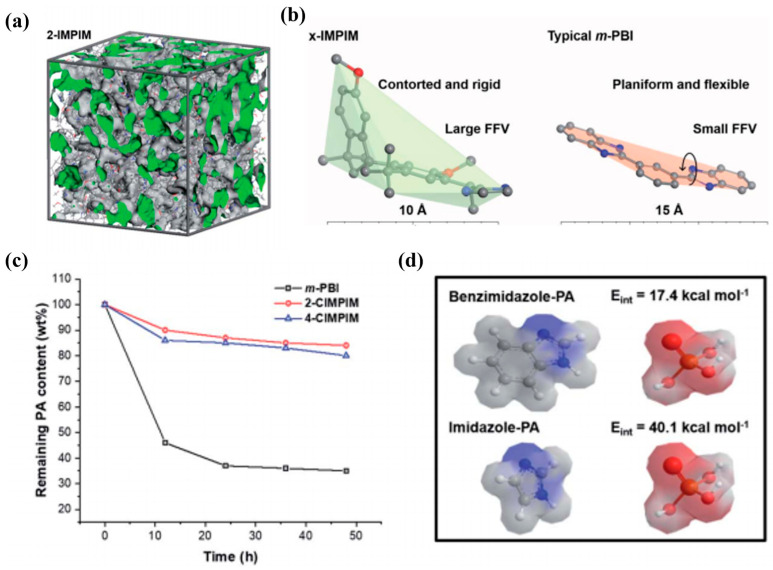
(**a**) The simulated amorphous cells of 2-IMPIM; (**b**) bat models for x-IMPIM and typical m-PBI; (**c**) the PA-retaining ability of 2-CIMPIM, 4-CIMPIM, and m-PBI at 80 °C and 40% RH; (**d**) the interaction energy between Im (or benzimidazole) and PA. Reproduced with permission from ref. [138]. Copyright 2021, Royal Society of Chemistry.

In Zhou’s work [138], a nitrogen-heterocyclic microporous polymer with well-organized, interconnected, and nanoscale channels was designed (consisting of contorted and rigid methoxylated spirobisindane moieties and methylated nitrogen heterocyclic linkages). Extensive and interconnected nanoscale channels were constructed by combining alkaline nitrogen heterocycles and inefficient chain filling in the x-IMPIM membrane (where x represents the aldehyde site on the Im rings), as shown in Figure 18. The intrinsic microporosity endowed x-IMPIM with a higher high-temperature proton conductivity and PA-retention ability than those of typical PBI.

Harilal [141] proposed a class of 3D iptycene-based ladder-like porous pyridine-bridged oxypolybenzimidazoles (IPyPBIs) with inherent but well-ordered micro-pores (about 12.1 Å). The IPyPBI membranes were found to trigger multiple hydrogen-bonding interactions with PA molecules within the micro-pores, leading to excellent PA retention at 160 °C. NPBI/PIM-BM-x membranes were prepared by introducing bromomethylated polymers (PIM-BM) with inherent micropores into NPBI by Guo et al. [142]. The microporous effect of PIM and the tightly cross-linked mesh structure contributed to higher PA retention, limiting PA leaching, and the PA-doped NPBI/PIM-BM-15 membrane exhibited 57.73% PA retention after 120 h at 80 °C/40% RH.

Tang’s team [143] proposed PA-doped intrinsically ultramicroporous membranes constructed from rigid, high-free-volume, Tröger’s base-derived polymers with an average ultramicropore radius of 3.3 Å. The membranes exhibited impressive proton conductivity and showed high retention of PA under highly humid conditions due to the delocalized effect and syphoning effect. Moreover, the resulting PA-doped PEMFC could operate across a broad temperature range from −20 °C to 200 °C, exhibited 95% peak power density retention after 150 startup/shutdown cycles at 15 °C, and was capable of completing more than 100 cycles even at −20 °C. This provides a new strategy for addressing the challenges of low-temperature operation and cold start in HT-PEMFCs.

### 3.7. Formation of Micro-Phase Separation

The design of both acidophilic and acidophobic groups on the polymer chain enables the formation of PEMs with micro-phase separation after the adsorption of PA [144]. The acidophilic phase facilitates the formation of continuous proton transport channel and enhances proton conductivity, while the acidophobic phase preserves the inherent mechanical properties of the material. Furthermore, the formation of the acidophilic phase enhances the interaction between the membranes and PA, leading to a reduction in acid loss.

**Figure 19:**
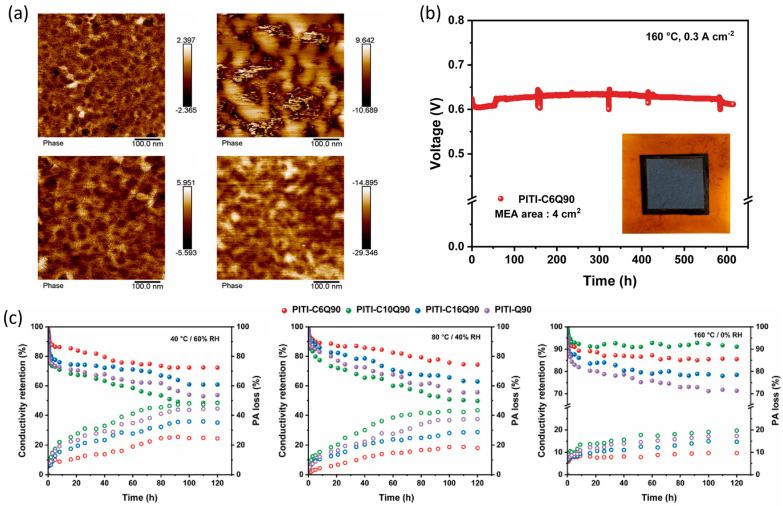
(**a**) AFM phase images of PITI-CmQ90 containing different aliphatic side-chains; (**b**) long-term durability for HT-PEMFCs using PA/PITI-C6Q90 membranes at 160 °C and 300 mA cm^−2^ constant current density; (**c**) the conductivity retention and PA loss of PITI-CmQ90 at different temperatures and humidities. Reproduced with permission from ref. [145]. Copyright 2023, Elsevier.

Lv et al. [145] fabricated PITI-CmQ90 membranes with a micro-phase separation structure by incorporating a flexible long aliphatic side-chain into a polymer matrix. They concluded that the doping of hydrophobic long aliphatic side-chain induced significant micro-phase separation, resulting in the formation of wider proton transport channels (as shown in Figure 19). The moderate ion aggregation within the microphase-separated PEM matrix leads to excellent cell durability and PA retention capacity via improved interaction with PA.

Zhang’s team [146] designed and synthesized Im-functionalized polyphenylene oxide (PPOs) with the objective of overcoming the leaching of PA and plasticizing of PA-doped PEMs by varying the grafting length of the side chains for size-controlled PA space confinement. They discovered that PPO-g-Az-x (where x represents the number of carbon atoms between the triazole and Im groups) outperformed OPBI in most of the key properties, including mechanical properties, PA retention ability, and proton conductivity, demonstrating that this strategy can trap PA molecules in the side-chain acidophilic micro-phase to enhance the PA retention and mitigating the plasticizing effect of PA on the polymer backbone. Another strategy to create and control micro-phase separation structures by tuning the conformation of the polymer backbone was reported [147]. A series of PTP-xm (where xm denotes the percentage of m-terphenyl in the backbone) were prepared by changing the ratio of the meta-to para-terphenyl units. Among the membranes, the PA-doped PTP-20 m membrane had a moderate micro-phase separation structure and exhibited the best PA retention ability, ideal fuel cell performance (1, 462 mW cm^−2^ at H_2_/O_2_), and good durability (300 h at 160 °C and 200 mA cm^−2^). The m-TPN-TMA/PA membranes were prepared by reacting trimethylamine (TMA) with precursor polymers [148]. The cationic groups acted as trapping sites for PA and formed ion-pair interactions with PA, which promoted the formation of different micro-phase separation structures. It was found to exhibit significant PA uptake and showed excellent stability (0.450 mV h^−1^) when tested at 160 °C for 100 h.

In Lu’s work, they prepared PPB and PPT (a family of poly (arylene piperidine) s) as the PEMs from triphenyl or biphenyl and N-methyl-4-piperidone [149]. The hydrophilic micro-phase of the piperidine ring groups and the hydrophobic micro-phase from the polymer backbone led to the PA-doped PPT and PPB membranes exhibiting excellent proton conductivity at 180 °C, and they were able to maintain stability for 1600 h at constant current densities of 120 mA cm^−2^ and 150 °C. Furthermore, they proposed an alternative approach to the fabrication of microphase-separated structures, employing atom transfer radical polymerization (ATRP) of a poly(1-vinylimidazole) (PVIm)-grafted polysulfone (PSf) membrane (P–g–V) [150]. These membranes demonstrated a sustained proton conductivity over 110 h under the cells test conditions.

A series of phosphonic acid- and imidazolium-containing polymer (PICP) ionomer membranes were designed by Meng’s team, in which the acidophilic (imidazolium) and acidophobic groups (–PO_3_H_2_) can be induced to form a microphase separation structure by PA [151]. The acidophilic phase facilitates the formation of continuous proton transport channels, and the hydrophobic phase not only synergistically conducts protons but also largely maintains the intrinsic mechanical properties of the material. The fuel cell assembled from the ionic membranes exhibited a peak power density of 493 mW cm^−2^ and a voltage decay rate of 50 μV h^−1^ in 100 h operation. An ether-free QPAF-4 membrane was investigated for HT-PEMs [144], which was a copolymer containing perfluoroalkylene and fluorenyl groups with pendant ammonium groups. The PA-doped membranes exhibited high proton conductivity and excellent PA retention at relatively low PA doping levels due to the inherent microphase-separated structure of the QPAF-4 matrix and the strong QA–PA interactions.

**Figure 20:**
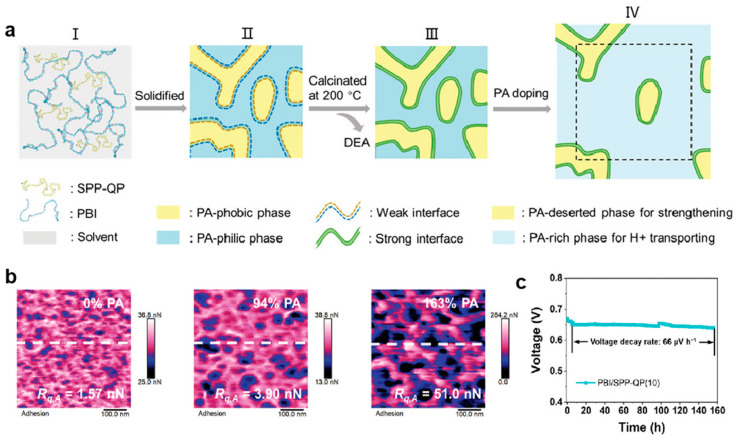
(**a**) Morphology assembly and interface evolution of the co-continuous phases in the PBI/SPP-QP membranes; (**b**) adhesion mapping results of PBI/SPP-QP(10) with different PA uptakes; (**c**) durability test results of PA-doped PBI/SPP-QP(10) at 140 °C. Reproduced with permission from ref. [152]. Copyright 2024, John Wiley and Sons.

Recently, a polyphenylene-based SPP-QP ionomer (a copolymer containing sulfonated p-phenylene, unsubstituted p-, and m-phenylene groups) was incorporated into PBI to obtain a PBI/SPP-QP composite membrane with micro-phase separation structure [152], as shown in Figure 20. SPP-QP, as intrinsic protonic conductor, has been shown to promote micro-phase separation and to contribute to the construction of the co-continuous PA-rich phase (PBI) and PA-deserted phase (SPP-QP). The formation of a strongly interacting co-continuous phase interface within the composite membranes leads to excellent cell performances with a peak power density of up to 719 mW cm^−2^ at 160 °C and a durability of more than 150 h.

## 4. Conclusions and Perspectives

In the current conditions of growing climate change concerns and technological advances [153], the hydrogen industry is expanding and fuel cells have greater potential in line with the concept of sustainable development. Due to the limitations observed in LT-PEMFCs, such as the limited CO tolerance, complex water and heat management, and sluggish reaction kinetics, there has been a greater focus on HT-PEMFCs. The high proton conductivity of PA under anhydrous conditions makes it a crucial component in HT-PEMFCs. In PA-doped HT-PEM, the ionic conductivity in the PEM depends largely on the PA content; on the other hand, blindly increasing the PA filling around the polymer chains leads to low mechanical properties of the PEM and accelerated leaking rate of PA, consequently deteriorating the electrochemical performance of the fuel cells. Therefore, reducing the leaching of PA is a significant area of research with the objective of enhancing the performance of HT-PEMFCs. Various efforts such as (i) designing crosslinked structures, (ii) incorporation of hygroscopic nanoparticles, (iii) improving the alkalinity of polymers, (iv) covalently linking acidic groups, (v) preparation of multilayer membranes, (vi) constructing microporous structures, and (vii) formation of micro-phase separation, have been made to mitigate PA leaching from the HT-PEMs. While promising successes in improving the performances of HT-PEMFCs have been achieved with the discussed strategies, challenges remain prevalent in developing new HT-PEMs with excellent and stable high-temperature proton conductivity as well as good mechanical strength in the future to meet the requirements of practical application of HT-PEMFCs.

Through understanding the proton-conducting mechanism of PA-doped HT-PEMs and drawing on the aforementioned strategies for alleviating PA leaching, we summarize the pros and cons of the strategies adopted previously and propose the prospects for future research directions as follows:

(1) Designing a cross-linked structure is recommended because cross-linking can substantially enhance the dimensional stability and mechanical properties of the PA-doped membranes. However, the cross-linking reaction tends to consume the basic sites on the main chain, leading to lower PA doping levels and reduced proton conductivity. One potential solution to this issue is to select crosslinkers which are rich in functional groups that promote proton conductivity (groups with basic sites, etc.).
(2) The incorporation of hygroscopic or layered inorganic materials into the polymer matrix can facilitate the formation of hydrogen bonds or intermolecular interactions with PA, thereby increasing the number of “trapping sites” for PA within the composite membranes. This strategy can enhance proton conductivity, mechanical properties, and acid retention. Nanoscale and agglomeration-free dispersion is the key point to maximizing the positive effect of the inorganic fillers.
(3) The introduction of more alkaline groups such as QA and Im can provide more sites for immobilizing PA within the membrane and reducing its leaching. However, improving the polymer’s alkalinity may also increase the ADL of the polymer membranes, leading to a decrease in mechanical stability and PA retention ratio, and therefore must be carefully controlled and balanced.
(4) Incorporating covalently linked organic acid groups (like –PO_3_H_2_) to replace part of the free PA is an effective way to alleviate PA leaching while maintaining decent proton conductivity. Nevertheless, covalently linked organic acid groups are not as effective as PA in proton conduction, so the amount of the covalently linked organic acid groups and doped PA should be well controlled. In addition, the membrane should have good mechanical strength so as to compensate for the inferior ion conductivity by reducing its thickness.
(5) Preparation of PEMs with a multilayer structure encapsulating PA in the core layer of the membrane has demonstrated the potential to mitigate PA leaching and improve durability. Further in-depth research is needed on the regulation of the composition and structure of the inner and outer layers, as well as the preparation process of the membrane, to meet the requirements of commercial applications.
(6) The development of PEMs with microporous structures can take advantage of the siphon effect to reduce acid loss without sacrificing proton conductivity. However, this type of microporous membrane only achieves the mechanical strength threshold necessary to be used as HT-PEMs, and there is much room for improvement.
(7) Designing and preparing a membrane with a similar microphase separation structure to Nafion is considered to be the most promising pathway for the development of high-performance and long-life low-PA-doped HT-PEMs. The mainchain should be a chemically and thermally stable structure which acts as the acid-phobic phase to provide intrinsic mechanical strength, while the side chain should be flexible and contain an alkaline functional group, which acts as the acidophilic phase to provide a continuous proton transport channel with a low PA doping level. To achieve this goal, more efforts need to be taken to explore new monomers and polymerization routes.
(8) Multiple strategies should be simultaneous applied to obtain HT-PEMs with excellent comprehensive performance. For example, multilayer structure can be designed with a high PA-doped polymer as the core layer and a very thin layer of PA blocking layer as the outer layer, where the core layer can be crosslinked PBI (with a basic group containing reagents as crosslinker), providing high proton conductivity and mechanical strength, while the outer layer can be a microporous-structured polymer or polymers with covalently linked organic acid groups or alkaline polymer/layered nanosheets nanocomposites that can provide a certain ion conduction and good PA retention ability.

## Figures and Tables

**Table 1:** Some polymer electrolyte membranes covalently linking acidic groups.

Membrane	Proton Conductivity (S·cm^−1^)	Peak Power Density (mW·cm^−2^)	PA/Cell Durability
OPBI/MDP-20%	1.4 × 10^−1^ (180 °C)	-	46% (PA retention tested after 0.5 h at 100 °C under water vapor)
OPBI/PTSA-20%	1.7 × 10^−1^ (180 °C)	-	67% (PA retention tested after 0.5 h at 100 °C under water vapor)
OPBI/CSA-20%	2.8 × 10^−1^ (180 °C)	-	73% (PA retention tested after 0.5 h at 100 °C under water vapor)
PBI/SPAEK-SPOSS-1%	1.1 × 10^−1^ (180 °C)	300 mW cm^−2^ (160 °C)	52% (PA retention tested after 9 h under 80 °C and 40% RH)
s-PBI	1~2.5× 10^−1^ (180 °C)	~600 mW cm^−2^ (180 °C)	30 μV h^−1^ (at 160 °C and 200 mA cm^−2^ over 3000 h)
c-mPBI/CeTOPT (50)	1.25 × 10^−1^ (180 °C, 100% RH)	-	proton conductivity retention rate of 95.4% (after 48 h of water washing)
PBI-NH_2_-EPA-15	6.2 × 10^−2^ (170 °C)	-	~65% (PA retention tested after 240 h at 70 °C and 60% RH)
PPF/50PBI	6.2× 10^−2^ (140 °C)	607 mW cm^−2^ (160 °C)	proton conductivity retention rate of 95.22% (after 20 h at 120 °C)
P/PITP-20	9.9 × 10^−2^ (140 °C)	812 mW cm^−2^ (180 °C)	0.45 mV h^−1^ (at 140 °C and 150 mA cm^−2^ over 100 h)

**Table 2:** Some multilayer membranes.

Membrane	Cell Activity	Durability
Asymmetrically porous PBI	835 mW cm^−2^ (160 °C)	0.283 mV h^−1^ (150 °C and 200 mA cm^−2^) ^1^
GMF/mPTFE	614 mW cm^−2^ (140 °C)	27.2 mW cm^−2^ h^−1^ (140 °C and 0.4 V) ^2^
Three-layer membrane	-	2.3 μV h^−1^ (180 °C and 200 mA cm^−2^) ^1^
Membrane (with a leaf- like three-layer porous structure)	713.6 mW cm^−2^ (160 °C)	0.73 mV h^−1^ (160 °C and 200 mA cm^−2^) ^1^
*p*-OPBI-ATMP/PA	980 mW cm^−2^ (160 °C)	5.46 µV h^−1^ (160 °C and 200 mA cm^−2^) ^1^

^1^ Voltage decay rates. ^2^ Power density decay rates.

## Data Availability

Not applicable.

## Glossary of Abbreviation

		OPBI	Poly (aryl ether benzimidazole)
		PA	Phosphoric acid
Abbreviation Description		PAEK	Poly (arylene ether ketone)
ADL	Acid doping level	PAM	Polyacrylamide
AST	Accelerated stress test	PANI	Polyaniline
BOPBI	Branched poly (aryl ether benzimidazole)	PBI	Polybenzimidazole
Br-HPP	Bromomethylated poly(p-xylene)	PBI-O-PhT	3,3-bis(p-carboxyphenyl)phtalide
Ce-TOPT	Cerium triphosphonicisocyanurate	PEM	Proton exchange membrane
CL	Catalyst layer	PEMFCs	Proton exchange membrane fuel cells
CMBelm	2-Chloromethyl benzimidazole	PGO	Phosphonated graphene oxide
CMPSf	Chloromethyl polysulfone	PICP	Phosphonic acid- and Imidazolium-containing polymer
CSA	Camphorsulfonic acid	PIL	Poly (ionic liquids)
GDL	Gas diffusion layer	PIMs	Polymers of intrinsic microporosity
GO	Graphene oxide	PPF	Phosphonated phenolformaldehyde
GTA	Glycidyl trimethyl ammonium chloride	PPO	Poly (2,6-dimethyl-1,4-Phenylene Oxide)
HCCP	Hexachlorocyclotriphosphonitrile	PTFE	Polytetrafluoroethylene
HT-PEMFCs	High-temperature proton exchange membrane fuel cells	PTSA	P-Toluenesulfonic acid
Im	Imidazole	PVIm	poly(1-vinylimidazole)
ImCCP	Imidazole—chlorocyclotriphosphonitrile	PVP	Poly (vinyl pyrrolidone)
IPN	Interpenetrating polymer network	PWA	Phosphotungstic acid (H_3_PW_12_O_40_·nH_2_O)
IPyPBIs	Iptycene-based ladder-like porous Pyridine-bridged oxypolybenzimidazoles	QA	Quaternary ammonium
KH560	3-glycidoxypropyltrimethoxysilane	SBA	Santa barbara amorphous-15
LT-PEMFCs	Low-temperature proton exchange membrane fuel Cells	SiC	Silicon carbide
MDP	Mono-n-dodecyl phosphate	SiO_2_	Silica
MEA	Membrane electrode assembly	TiO_2_	Titanium dioxide
Mus	Muscovite (KAl_2_(Si_3_Al)O_10_(OH)_2_)	TMA	Trimethy-lamine
NbPBI	Norbornene-type polybenzimidazole	Verm	Vermiculite
NMBA	N, N’-(methylene) bisacrylamide	ZrO_2_	Zirconia
